# *Kirigami* triboelectric spider fibroin microneedle patches for comprehensive joint management

**DOI:** 10.1016/j.mtbio.2024.101044

**Published:** 2024-04-04

**Authors:** Shuhuan Li, Suwen Cao, Huihui Lu, Bingfang He, Bingbing Gao

**Affiliations:** College of Biotechnology and Pharmaceutical Engineering, School of Pharmaceutical Sciences, Nanjing Tech University, Nanjing, 211816, China

**Keywords:** *Kirigami*, Spider fibroin, Microneedles, TENG, Osteoarthritis, Wounds

## Abstract

Joint injuries are among the leading causes of disability. Present concentrations were focused on oral drugs and surgical treatment, which brings severe and unnecessary difficulties for patients. Smart patches with high flexibility and intelligent drug control-release capacity are greatly desirable for efficient joint management. Herein, we present a novel *kirigami* spider fibroin-based microneedle triboelectric nanogenerator (KSM-TENG) patch with distinctive features for comprehensive joint management. The microneedle patch consists of two parts: the superfine tips and the flexible backing base, which endow it with great mechanical strength to penetrate the skin and enough flexibility to fit different bends. Besides, the spider fibroin-based MNs served as a positive triboelectric material to generate electrical stimulation, thereby forcing drug release from needles within 720 min. Especially, *kirigami* structures could also transform the flat patch into three dimensions, which could impart the patch with flexible properties to accommodate the complicated processes produced by joint motion. Benefiting from these traits, the KSM-TENG patch presents excellent performance in inhibiting the inflammatory response and promoting wound healing in mice models. The results indicated that the mice possessed only 2% wound area and the paw thickness was reduced from 10.5 mm to 6.2 mm after treatment with the KSM-TENG patch, which further demonstrates the therapeutic effect of joints in vivo. Thus, it is believed that the proposed novel KSM-TENG patch is valuable in the field of comprehensive treatments and personalized clinical applications.

## Graphical abstract

**Figure:**
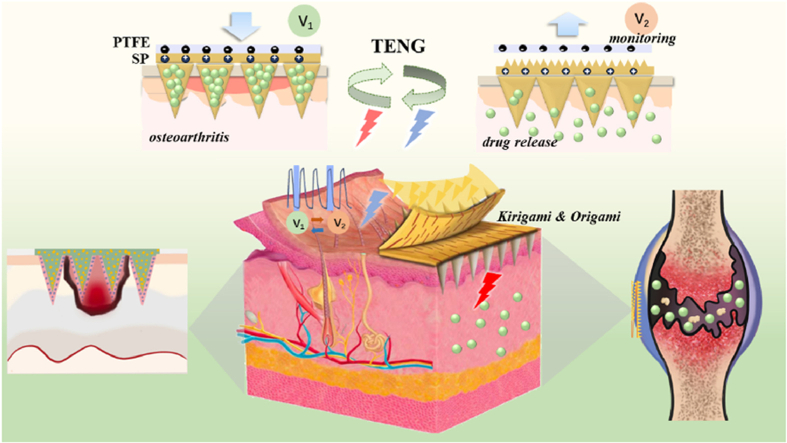

## 1 Introduction

Generally, joint injuries are caused by many factors such as accidents, repetitive stress, exercise, osteoarthritis (OA), and trauma, which bring mental adverse effects and physical trauma to patients. Among them, joint wounds and OA are one of the leading causes of disability. Wounds on joint areas are inevitably stretched in frequent bending and movements, which causes inconvenience of effective wound closure, and remains non-healing and inflamed for long periods, thus forming chronic infections. Besides, OA is a global degenerative osteoarthropathy mainly characterized by chronic synovial inflammation and articular cartilage degeneration, which leads to serious endangering of the joints and induces tissue damage, hypertrophy, and even death [[1], [2], [3], [4]]. Currently, joint management extensively focuses on the control of disease progression by drug and surgical treatment [[5], [6], [7], [8], [9]]. However, injections may induce swelling and lead to poor patient acceptance and compliance. In addition, it was reported that the frequent use of drugs has the potential risk of inducing acute kidney injury and damaging the gastrointestinal tract due to its toxic side effects and large drug dosage. To address these issues, transdermal drug delivery patches have been devoted to offering a potential alternative administration method for joint treatment. Although with numerous breakthroughs, most of the existing patches have difficulty in realizing efficient and long-term drug release. Additionally, they are not flexible enough to fit the special stretch of the joint in complicated movement processes. Therefore, it is highly anticipated to develop a novel transdermal patch with flexible properties and an efficient release rate for joint treatment.

**Fig. 1:**
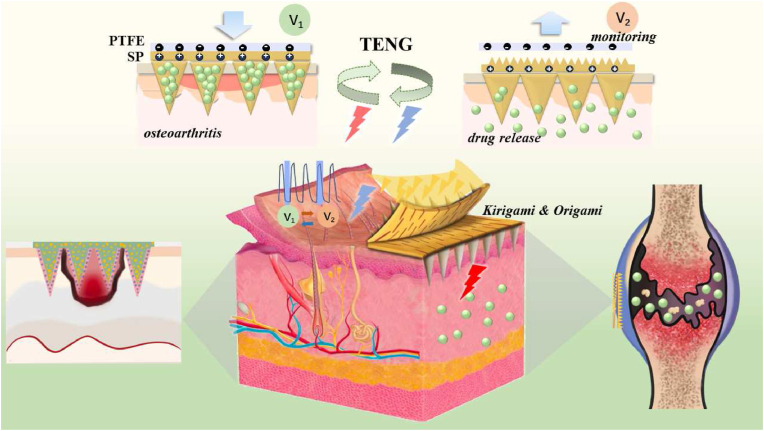
*Kirigami* triboelectric spider fibroin microneedle patches for comprehensive joint management.

Herin, we propose a *kirigami* spider fibroin microneedles triboelectric nanogenerator (KSM-TENG) with promising multifunctional properties for joint treatment, as shown in the schematic in Fig. 1. Transdermal drug delivery systems have shown excellent potential in avoiding the first-pass effect and maintaining blood levels to produce a sustained release effect; however, the delivery efficiency is commonly limited due to the barrier function of the stratum corneum (SC). Generally, SC is 10–40 μm in thickness. Different from traditional drug delivery systems, microneedles (MNs) consist of microscale needles, usually with lengths from 600 to 800 μm, which can penetrate the SC barrier without causing pain or damaging nerves or blood vessels, achieving minimally invasive treatment and improving patient acceptability [[10], [11], [12], [13], [14], [15], [16]]. Meanwhile, electroporation (EP) has also proven to be a convenient and effective nonpharmacological intervention to deliver charged and hydrophilic through the skin. As first reported by Zhonglin Wang [17], triboelectric nanogenerators (TENGs) serve as sustainable energy harvesting sources because they can harvest mechanical energy by human movement and convert it into electrical energy, which provides a feasible option for transdermal drug delivery through high-voltage electric pulses [[18], [19], [20]]. In addition, *kirigami* are also introduced in the microneedle patch triboelectric nanogenerator to avoid stretchability limitations in vivo [[21], [22], [23], [24], [25], [26], [27]]. By means of *kirigami* structures, the whole flat structure can be transformed into a three-dimensional structure under external strain, allowing for sufficient conformability and stretchability to accommodate complex motion processes [23,26,28]. Moreover, the motions of joints can be monitored through relative voltage changes of the triboelectric nanogenerator device. Such multifunctional transdermal patches integrated with the advantages of *kirigami* structures, MNs, and TENGs devices hold dramatic prospects for intelligent joint treatment. However, research on this combination is still insufficient.

In this paper, we present a *kirigami* spider fibroin-based MN patches and a triboelectric nanogenerator device for drug delivery (Fig. 1). The MN patch was first replicated from a negative silicone rubber (Ecoflex) mold engraved by a laser. Notably, the Ecoflex molds were biaxially prestretched and poured with MXene solution before laser engraving. After recovery, the mold with patterned conical grooves and the MXene surface changed from cracked to stacked. Then pouring the precursor, the spider fibrion-based MNs patch was eventually fabricated with superfine patterns and wrinkled surfaces. Based on such a simple manufacturing process, MNs patches can be fabricated easily, precisely, and on a large scale. Typically, spider fibrion (SP) is a natural material with great biocompatibility and possesses enough mechanical properties to satisfy the requirement of skin penetration [[29], [30], [31]]. Meanwhile, SP has a strong tendency to donate electrons, which can be used as a positive material and impart charge generation upon frictional contact with negative materials, thus realizing the efficient drug release achieved by electrical stimulation. It is worth mentioning that the origami and *kirigami* structures endowed the KSM-TENG patch with flexible properties, which contributed to not only fitting the complicated movement processes but also improving the mechanical behavior of friction. Taking advantage of these capacities, the results demonstrated that the KSM-TENG could exhibit excellent therapeutic performance in treating mice. Therefore, it is believed that the proposed KSM-TENG patch with these features will provide a prospective development for drug-delivery systems and clinical applications.

## 2 Results and discussion

### 2.1 Fabrication of the KSM-TENG patch

**Fig. 2:**
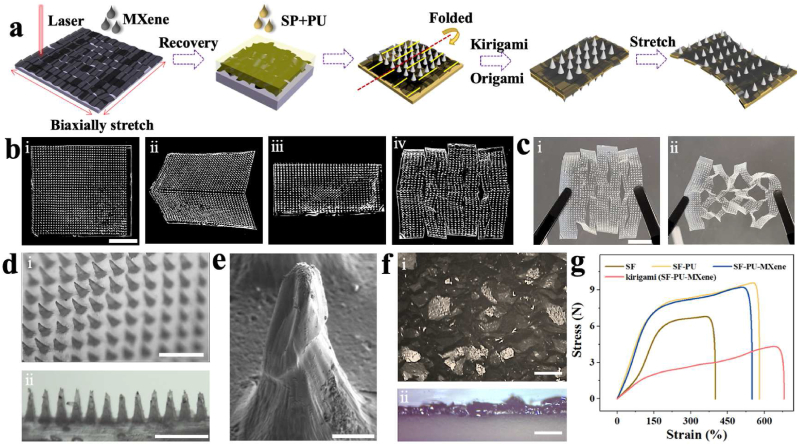
Fabrication and characterization of the SP-MXene-based MN film. (a) Schematic view of the fabrication of SP-MXene-based MN films. (b) Micrograph of the SP-MXene-based MN film after *kirigami*: (i) primitive film; (ii) folding; (iii) folded; (iv) cutting. Scale bar: 1 cm. (c) Microimages of the SP-MXene-based MN film after being stretched: (i) nonstretched; (ii) stretched. Scale bar: 1 cm. (d) Micrograph of the MNs: (i) vertical view; (ii) side view. Scale bar: 2 mm. (e) SEM images of the MNs. Scale bar: 200 μm. (f) Micrograph of the stacked MXene-based film: (i) front view; (ii) side view. Scale bar: 400 μm. (g) Mechanical characteristics of SP-MXene-based MN films with different materials and *kirigami* structures.

The fabrication process of the KSM-TENG patch with origami and kirigami structures is schematically presented in Fig. 2a. Typically, SP have been used for MN fabrication because they possess enough mechanical strength to satisfy the hardness requirements and exhibit puncture ability. However, despite the notable mechanical strength, the low flexibility has great difficulty fitting the long-term dymataic motion. In addition, motion sensing exhibits better performance on a flexible base, contributing to its excellent stretch-recovery properties. To solve this issue, polyurethane (PU) and *kirigami* structures were employed in this system to fabricate the flexible layer of the KSM-TENG patch. Moreover, MXene, known as a two-dimensional material with high conductivity, is generally limited because of its rapid crack propagation. Therefore, a closely packed MXene layer could be fabricated by prestretching. Specifically, the Ecoflex molds were biaxially stretched before engraving, while conical holes with personalized patterns were formed by laser engraving, and then the surface was drop cast with MXene solution. After recovery, the SP solution was poured into the negative molds and degassed several times to generate the needle tips. Then, the SP-PU precursor (SP mixed with PU solution at a volume ratio of 7:3) was filled with negative molds to prepare a flexible base. After drying, the patch could peel off from the negative molds. In addition, the kirigami structure can stretch the initial mold into an extended state, which endows the patch with more flexible properties. Therefore, kirigami structures with individualized patterns are generally engraved by a laser machine at 10.5% power, which endows the patch with the ability to sustain large strain. In addition, oirigami generally involves paper folding from an initially flat state into a compact volume, which imparts the KSM-TENG patch with multiple functional integration in a limited size, including triboelectric stimulation drug release and motion sensing. Thus, a patch with high frictional conductivity, excellent mechanical strength, and palmary flexibility was inevitably obtained.

Taking advantage of the laser engraving process, the KSM-TENG patch with *kirigami* structures was fabricated, which showed uniform tensile properties and flexibility (Fig. 2b and c). In addition, as shown in Fig. 2d, the needle sizes are 0.8 mm in height and 0.4 mm in diameter, which exhibits uniform morphology and neatness of arrangement. Moreover, SEM images further demonstrate the complete and superfine microstructure of the MNs in Fig. 2e. The results indicate that MN patches with *kirigami* structures can be fabricated by a simple and high-precision engraving and stretch-recovery process. In addition, the stretch-recovery process is also introduced in this system to overcome the rapid crack propagation of MXene. After recovery and drying, the cracked surface of MXene-based materials can be transformed into a wrinkled film, which greatly improves the conductivity (Fig. 2f). To evaluate the mechanical properties of the KSM-TENG patch, tensile limit tests were applied in vitro. The patches made of 100% SP solution, SP-PU mixed precursor, SP-PU-MXene mixed precursor, and SP-PU-MXene mixed precursor with kirigami-origami structures demonstrated that the flexible performance of the KSM-TENG patch with *kirigami* structures was significantly improved (Fig. 2g).

### 2.2 The working mechanism of the KSM-TENG patch

**Fig. 3:**
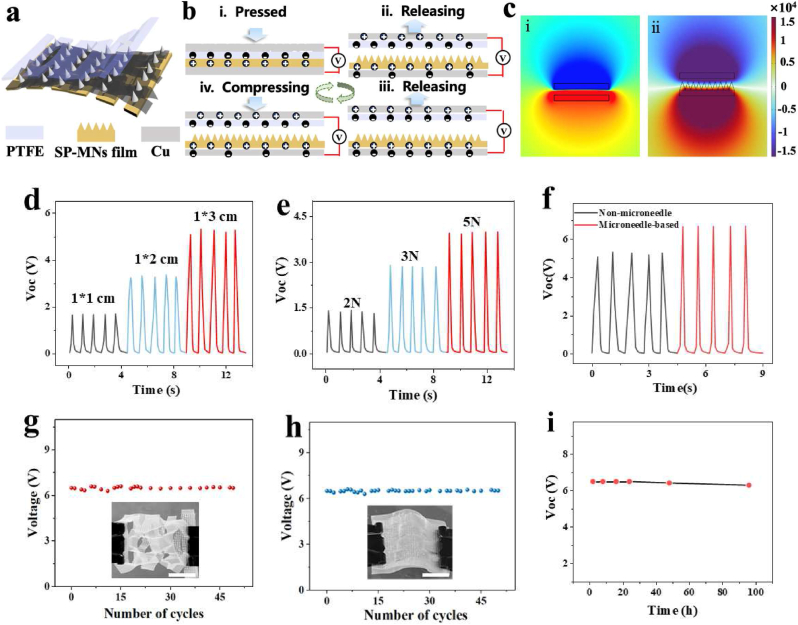
Structural design, working mechanism, and output performance of the KSM-TENG. (a) The structure of the KSM-TENG based on the SP-MNs film, PTFE and Cu. (b) Schematic view illustrating the working principle of the KSM-TENG under the vertical contact-separation mode. (c) Simulation potential diagrams of the KSM-TENG by COMSOL software: (i) without a needle structure and (ii) with a needle structure. Generated output characteristics under various conditions: (d) with different areas, (e) with different input forces, and (f) with different surface structures. Stability test of the KSM-TENG: (g) under the stretched state and (h) under the bent state. (i) The durability demonstrated by a 100 h long-term test. Scale bar: 1 cm.

To date, several synthetic polymers, such as polyimide (PI), polyethylene terephthalate (PET), and Kapton, have been introduced into TENGs to serve as triboelectric materials [[32], [33], [34]]. However, long-term wearing devices need better biocompatibility, flexibility, and skin comfort. As a natural polymer, SP is classified as a positive triboelectric material that not only possesses a strong ability to lose electrons but also preferably has flexible and stretchable properties to attach to joints and capture the corresponding electrical motion sensing signal. The design of the KSM-TENG is shown in Fig. 3a. SP and PTFE constitute the positive and negative triboelectric pair, respectively, and Cu tape is attached on them employed as bottom-positive and top-negative layers.

The KSM-TENG works in the vertical contact-separation mode, and the working mechanism is illustrated in Fig. 3b. Specifically, when other external compressive forces are in contact with the KSM-TENG, the PTFE film can generate a negative charge and displace vertically toward the SP layer, resulting in opposite surface charges and accumulating an equal number of positive and negative charges due to the triboelectric effect (Fig. 3b–i). Once the external force is slowly released, the PTFE recovers to its original state through the internal mechanical force. Thus, an electrostatic potential difference is generated when the top layer (i.e., PTFE) and the bottom layer (i.e., SP) begin to separate, resulting in electrons flowing from the top to bottom Cu electrodes (Fig. 3b–ii). When the triboelectric layers completely separate and recover to their initial positions, the electrical potential difference no longer increases and is in equilibrium with triboelectric charge distributions (Fig. 3b–iii). Subsequently, once the external compressive force is reapplied to the KSM-TENG, the pressing step makes the two triboelectric layers start to move in the direction of contact with each other, which leads to the generation of a reverse electrical potential difference from the bottom electrode to the top Cu electrode (Fig. 3b–iv). Therefore, an alternating electrical output current is generated by cycles of pressed/released compressive forces, which can be stored and applied as an external power device. In addition, it is demonstrated that the contact surface area is an essential parameter for charge separation. As shown in Fig. 3c, to verify the influence of the SP film with different surface contact areas, the electrical potential difference was theoretically studied by COMSOL Multiphysics in two states: non-MN structures (Fig. 3c–i) and with-MN structures (Fig. 3c–ii). The results demonstrated that the electrical potential distribution across the KSM-TENG with MN structures is significantly higher than that of the flat SP film. Such improved output performance is mainly attributed to the “bending–friction–deformation” behavior of MNs, which results in the enhancement of contact areas between triboelectric materials.

**Fig. 4:**
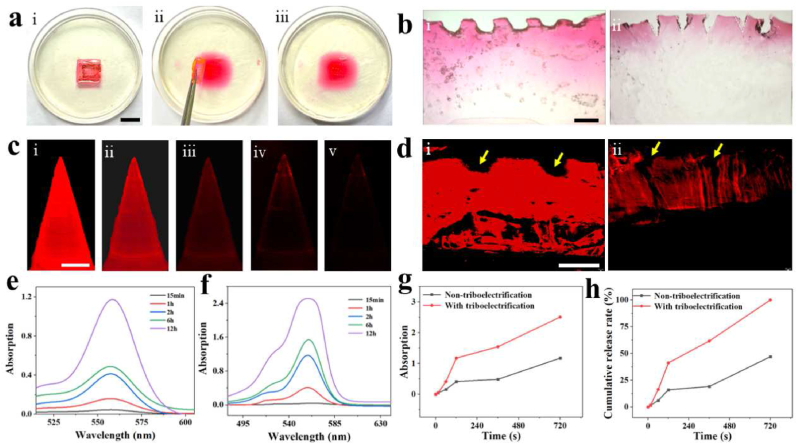
(a) Images of drug release into the hydrogel: (i) prerelease; (ii) release; (iii) after release. Scale bar: 1 cm. (b) Microimages of the comparison of drug release into the hydrogel over the same time: (i) with triboelectrification; (ii) without triboelectrification. Scale bar: 800 μm. (c) Fluorescence photos of drug release from SP-MNs with triboelectrification at (i) 15 s; (ii) 1 h; (iii) 2 h; (iv) 6 h; and (v) 12 h. Scale bar: 400 μm. (d) Fluorescence photos of the comparison of drug release into the hydrogel over the same time: (i) with triboelectrification; (ii) without triboelectrification. Scale bar: 800 μm. Absorption spectra over time: (e) nontriboelectrification (f) with triboelectrification. (g) Comparison of absorbance spectra between triboelectrification and nontriboelectrification over time. (h) Cumulative drug release under triboelectrification and nontriboelectrification over time.

To verify the electrical output performance of the KSM-TENG, the corresponding open-circuit voltage (Voc) under different conditions was measured and evaluated. As described in previous literature, the output voltage effect is generally influenced by the surface area, compression force, and surface roughness. Thus, the results indicated that with increasing area and pressing force, the Voc gradually increases from less than 2 V–4 V (Fig. 3d and e). Then, as shown in Fig. 3f, the SP film with MN structures reached the highest open-circuit voltage, which is attributed to the “bending–friction–deformation” effect expanding the contact-separation area, thus realizing a higher electrical output. Besides, due to the abundance of –F groups and oxygen-containing capping functional groups on the surface, MXene possesses excellent metal conductivity and a highly electronegative surface. Thus, as shown in Fig. S2, we also test the effect of the MXene on output performance, which demonstrates that MXene has high conductivity properties that significantly enhance output performance. Additionally, repetitive stretching and bending tests were introduced into the KSM-TENG to evaluate its flexibility and stretchability under an external force. Fig. 4g and h shows the Voc values under stretching–recovering and bending–relaxing conditions, indicating that the KSM-TENG showed a stable response during different cycles of mechanical deformation–relaxation. In addition, a 100 h long-term test was also performed to evaluate the durability, which demonstrated that the output performance did not degrade. These results indicated that the KSM-TENG can be employed for bioelectronic applications and offers a possibility for practical sensing.

### 2.3 *The drug release system of the KSM-TENG patch* in vitro

The transdermal drug delivery (TDD) system possesses several benefits over traditional oral and injectable modes of administration because it effectively avoids the first-pass effect, ensures stable and long-lasting blood levels, and improves patient compliance. MNs consist of micron-sized needles (usually less than 1 mm) capable of creating small holes and allowing more biomolecules and drugs to puncture the skin without reaching nerves. However, most drugs are water-soluble, with high molecular weight, and in dissociated form, which is limited to passive diffusion capacity. Thus, among the various methods for TDD, the MNs combined with EP have been proven to be strong candidates for permeation enhancement. Generally, EP involves creating transient pores of the plasma membrane by using high-voltage electrical pulses for cell introduction and percutaneous transport. With the assistance of electric current (usually less than 0.5 mA cm^−2^), the charge molecules can flow and result in the electroosmotic flow of biofluids to transport particle/biochemical ions and large protein molecules, thus facilitating the on-demand drug delivery [[35], [36], [37], [38], [39]]. Besides, TENG served as exogenous stimuli generally combining contact electrification and electrostatic induction, which are applied to harvest abundant kinetic energy from human mechanical motions and convert it into electricity to power in vivo EP. Moreover, the electric field powered by TENG can not only generate pores in the skin but also provide a localized driving force for the transport of hydrophilic molecules. Notably, TENG can convert almost 80% of biomechanical and environmental energy, which provides a promising method for energy harvesting and supplies stabilized voltage for drug delivery devices. Thus, among the suggested tools for TDD, the combination of MNs and TENGs has been considered an effective permeation enhancement method for molecular delivery.

By mimicking the origami structure, the KSM-TENG patch is divided into two sides: the top layer for friction charging and the bottom layer for drug delivery. MNs with a unique tip structure can penetrate the skin and are used as a promising drug carrier for transdermal drug delivery. To study the process of drug release, we utilized a gelatin hydrogel extracted from bovine skin as a model tissue because its optical transparency is similar to that of skin tissue. In addition, for visual monitoring, rhodamine B (RhB) integrated into MNs was chosen as a model to monitor the release process. Thus, cured gelatin was prepared, and MNs containing RhB penetrated through the surface of the hydrogel (Fig. 4a). In the diffusion test, PTFE was used for triboelectrification, and a pressure of 3 N was applied to the hydrogel surface. At various time points, the KSM-TENG patch was separated from the hydrogel, and the drug-diffused gelatin was immersed in the decolorizing solution to cease further release. After dipping in the decolorizing solution, the gelatin gradually swells in volume. Then, the absorbance of the decolorizing solution was measured by UV–Vis absorption spectroscopy to quantify the rate of the drug release process.

Continuous electrical stimuli are an essential propulsion for higher drug release. To confirm this, the experiment was divided into two groups: the KSM-TENG patch on gelatin with continuous contact-separation operation for 720 min and a control group under the same conditions without connecting the TENG. As shown in Fig. 4b and d, microscopy and fluorescence images presented distinct contrasts of the skin cross-sectional images, respectively. This demonstrated that more RhB was delivered into the skin when treated with a TENG-driven system, indicating the feasibility of an electrically assisted drug release system. In addition, as shown in Fig. 4c, the fluorescence images revealed a gradually decreased fluorescence intensity with increasing release time, indicating sustained release of drugs. Moreover, the absorbance of RhB at different periods was measured at 560 nm. As shown in Fig. 4e–g, all spectra exhibited low concentrations of RhB at 1 h, but the absorption of decolorizing solution after triboelectrification increased significantly after 1 h. To evaluate the release process, the cumulative release amounts were quantified by calculating the mean absorbance (Fig. 4h). Thus, these results suggested that the release of drugs could be promoted by triboelectrification.

### 2.4 Application of the KSM-TENG patch for motion sensing

In our daily lives, strenuous physical movements could severely limit controllable drug release. In particular, some special parts, such as joints, are inevitably stretched during dynamic movements, which could cause infection and hinder the healing process of disease [[40], [41], [42]]. For joint treatment, the granulation tissue would be stretched by frequent involuntary joint movements, which disrupts the normal healing process. Unfortunately, it is difficult for patients to discover the unconscious movements of joints and notify caregivers of medical staff, which leads to aggravation of the condition. Thus, monitoring the motion of joints is critical because it could directly indicate unconscious dynamic movements by easily converting external strenuous stretching into electrical signals, which also benefits timely notification to patients and their medical staff to intervene effectively [28,43,44]. Since TENGs can efficiently transform mechanical excitation into electrical signals, they have attracted great attention for self-powered motion sensing applications. For example, Dudem et al. [45] developed a silk-based TENG with high resistance to water solubility by employing an alcohol-annealing treatment. The alcohol post-treatment greatly improves the water-insolubility of silk, with usage times ranging from minutes to several hours, thus is expected to be used for skin wearable devices. Besides, Dong et al. [46] fabricated a wearable TENG by regenerating silk fibroin and carbon nanotube conductive film with high elasticity. The conductivity increased from 0 to 746 S/m with the increase of carbon nanotube content. Moreover, the electrical response produced by human movement is different during diverse types of human activities, which provides an ideal choice to serve as an epidermal self-powered sensor for multifunction human motion monitoring. Thus, the output voltage of the TENG can provide a feasible option to identify corresponding human movement and provide energy for disease treatment.

**Fig. 5:**
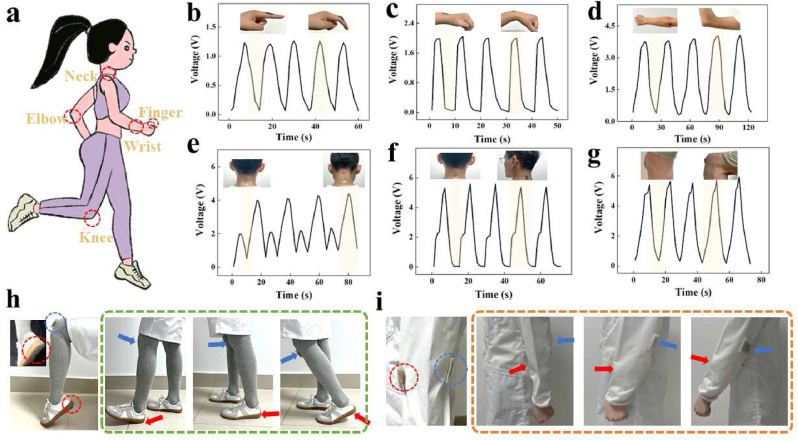
KSM-TENG patch vemployed for multifunction monitoring applications. (a) Schematic illustration of human activity. The relative voltage response of the (b) finger, (c) wrist, and (d) elbow bending. Output voltage response generated by (e) nodding and (f) twisting. (g) The voltage changes of the human joint attached to the knee. (h) The electrical energy generated during walking for knee therapy. (i) The electrical energy generated during arm friction for elbow therapy.

In this regard, a TENG device is employed by integrating SP and PEFE films with *kirigami* structures, which endow it with enough flexibility to fit the human body. To investigate the motion sensing ability of the TENG, such a flexible device was attached to different positions of the body (Fig. 5a). As shown in Fig. 5b–d, the KSM-TENG patch was located on the finger, wrist, and elbow. Then, the electrical power was generated by biomechanical movement of bending at different angles; thus, the corresponding voltage changes could be stably recorded and evaluated. In addition, the voltage changes of the nodding and twisting modes demonstrated that they possess sufficient sensing sensitivity for monitoring respiration (Fig. 5e and f). Additionally, in addition to monitoring small dynamic movements, the patch was placed on the knee to harvest signals upon bending–releasing behavior, thus obtaining the real-time responses at the joints (Fig. 5g). Except for motion sensing, the useful mechanical energy collected by the KSM-TENG patch could also provide electrical output for disease treatment. As shown in Fig. 5h and i, the KSM-TENG patches can be applied in different sites for therapeutic and sensing. Besides, the electrical energy generated during walking and arm friction used for knee and elbow joint therapy, respectively. Based on these results, the motion sensing of the human body can be clearly identified by converting the electronic response of the KSM-TENG patch, which provides a promising method for health information monitoring and mechanical energy collection.

### 2.5 Therapeutic effects of the KSM-TENG patch

#### 2.5.1 The efficiency of the KSM-TENG patch on joint wounds healing

**Fig. 6:**
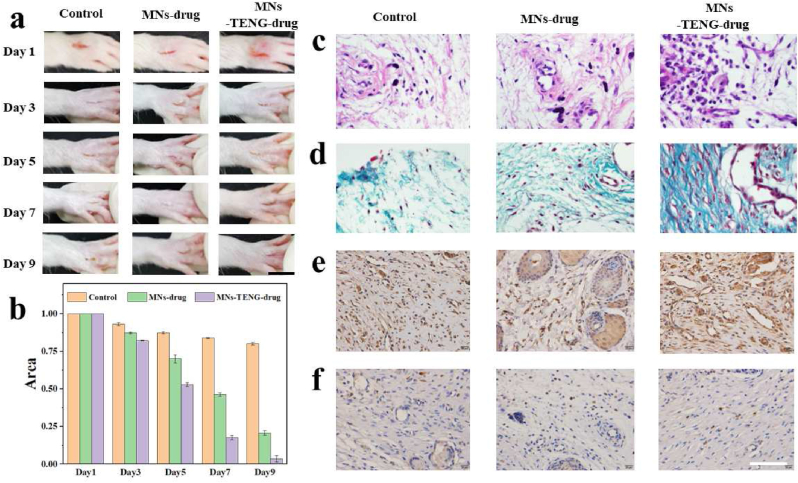
Evaluation of the MNs patches for joints wound healing. (a) Optical images of the skin wounds in three groups (control, MNs-drug, and MNs-TENG-drug) within 9 days. Scale bar: 1 cm. (b) Statistical analysis of wound area (%) (mean ± SD; n = 3). (c) H&E staining of skin wounds tissues. (d) Masson staining of skin wounds tissues. (e) Immunohistochemical staining of IL-6. (f) Immunohistochemical staining of IL-6. The scale bars are 100 μm in (d), (e), (f), and (g).

To further demonstrate the curative effect of the KSM-TENG patch for joints wound healing, 1 cm wounds on joints were created to produce a full-thickness rat model of infected skin defects. Meanwhile, the KSM-TENG patch (control group), S KSM-TENG patch encapsulated with hEGF (MNs-drug group), and KSM-TENG patch encapsulated with hEGF by electrical stimulation (MNs-TENG-drug group) were used for evaluating in vivo experiments. First, as revealed in the H&E image (Fig. S1), it can be seen that clear skin tissue fractures in mice after MN insertion, which demonstrates the MN patch could penetrate the epidermis well to reach deeper tissues. As shown in Fig. 6a and b, the changes of wounds were recorded and measured on days 1, 3, 5, 7, and 9 to evaluate the wound healing process. Generally, these photographs demonstrate that the wounds of the rats were essentially healed after 9 days in the MNs-TENG-drug group, which exhibits higher a closure rate than MNs-drug group and control group. When it comes to further explorations of wound healing processes, hematoxylin and eosin (H&E) staining and Masson staining should be conducted. From the results of H&E staining, it can be seen that the MNs-TENG-drug group showed regenerated granulation tissue at the wound and presented the most complete and thickest stratum corneum (Fig. 6c). Besides, Masson staining was conducted to exhibit the collagen deposition in the wound (Fig. 6d). The results demonstrated that the MNs-TENG-drug group showed accelerated growth of large numbers of fibroblasts and deposition of collagen in the injured tissue compared to other groups, thus demonstrating this system is conducive to skin recovery.

Except for the regenerated granulation tissue and collagen deposition, inflammation is another crucial part during the wound remolding, which could impede wound healing. Thus, immunohistochemical staining was carried out to analyze typical proinflammatory factors of interleukin-6 (IL-6) and tumor necrosis factor α (TNF-α). It can be seen that the higher expression quantity of IL-6 and TNF-α in the control group, indicates a serious inflammatory response during the wound healing process. In the MNs-TENG-drug group, almost no IL-6 and TNF-α secretion were detected, demostrating this system significantly accelerated the repair process (Fig. 6e and f). The notable anti-inflammatory effect and significant collagen deposition suggested that MNs-TENG-drug group have potential for joints wound healing applications.

#### 2.5.2 The efficiency of the KSM-TENG patch for OA treatment

**Fig. 7:**
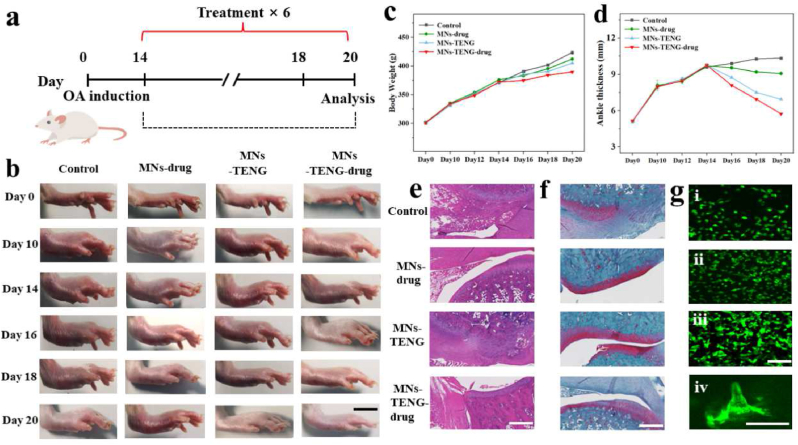
Therapeutic effect of the KSM-TENG patch in the ankle. (a) Schematic diagram for showing the therapeutic process of the KSM-TENG patch. (b) Photographs of the hind paw profile after 20 days. Scale bar: 500 μm. (c) Changes in body weight in different groups. (d) Changes in ankle thickness in different groups. (e) HE staining of the ankle tissue. Scale bar: 100 μm. (f) Safranin O-fast green staining of the ankle tissue. Scale bar: 100 μm. (g) Fluorescence images of CT 26 cells (i-iii) cultured on KSM-TENG patches for 1, 3, and 5 days and (iv) cultured on needle tips for 5 days. Scale bar: 100 μm. (For interpretation of the references to colour in this figure legend, the reader is referred to the Web version of this article.)

To evaluate the ability of the KSM-TENG patch to facilitate the OA treatment process, mice weighing approximately 300 ± 0.25 g were utilized as experimental models encapsulated with methotrexate (MTX). Specifically, we randomly assigned the mice into three groups: no treatment (control group), empty MN dressing with MTX (MNs-drug group), empty MN dressing with triboelectrification (MNs-TENG group) and MN dressing with MTX and stimulation by triboelectrification (MNs-TENG-drug group). As shown in Fig. 7a, we treated the mice for 6 times after 14 days and monitored their paw swelling daily. Thus, images of the healing process with different treatments on days 0, 10, 14, 16, 18, and 20 were captured. As shown in Fig. 7b, compared with the electrical stimulation therapy groups (MNs-TENG group and MNs-TENG-drug group), untreated OA mice maintained higher paw thickness due to joint inflammation. In addition, due to the avoidance of first-pass effects, the MN group exhibited better therapeutic efficiency than the control group. However, the therapeutic effect is still limited by the slow rate of drug release. As expected, the MN-TENG group exhibited the best curing effect because the release of drugs was facilitated by electrical stimulation, which showed that paw swelling almost disappeared after 6 days. Moreover, as shown in Fig. 7c and d, the body weight and ankle thickness were measured and analyzed, which further demonstrated that the MN-TENG group possesses an obviously lower swelling ratio, indicating a higher therapeutic effect than the other groups.

In addition, different staining tests were applied to further check the OA treatment effect. The skin tissue of the claw and ankle joint was stained with hematoxylin and eosin (H&E) to assess the OA levels. As shown in Fig. 7e, the control group exhibited typical OA symptoms, including vertical fissure, erosion denudation, and rough surface. In addition, a large number of proliferating synovial cells invade the joint cavity, which narrows the joint space and leads to decreased chondrocytes. Compared with the control group, the synovial tissue structure of the MN group of mice was relatively intact, and the joint space was enlarged. In contrast, the MN-TENG group showed better H&E staining results, and the ankle joint cavity was clear and free of impurities. Moreover, the integrity of articular cartilage is an important indicator of the response to OA treatment. From the Safranin O-fast green staining (Fig. 7f), it can be seen that the articular cartilage in the control group was dull in color, and the structure was gradually damaged. In addition, the articular cartilage of mice in the MN-TENG group was brightly and clearly stained with smooth and intact structures, indicating that the SP-MN-TENG patch is capable of facilitating the treatment of OA.

Notably, in vitro experiments were performed to demonstrate the cytocompatibility of the KSM-TENG patch (Fig. 7g). The patches were first immersed in cell culture medium; thus, their cytocompatibility could be demonstrated by CT 26 cells cultured in the leachate. By recording live/dead staining after 1, 3, and 5 days in culture, it could be observed that all CT 26 cells can be equally distributed on the patch, and almost no cell death was detected. All the results indicated that the KSM-TENG patch possesses satisfactory cytocompatibility.

## 3 Conclusion

In conclusion, inspired by *kirigami* structures, we fabricated an KSM-TENG patch with distinctive features for comprehensive joint treatment. To generate this MN patch, a laser-engraved template replication system was employed, during which MNs and stacked MXene surfaces were replicated from prestretched negative Ecoflex molds. The superfine structures imparted the MN patch with sufficient skin penetration during the long-term treatment of joint. Moreover, the SP-MNs were endowed with charge generation capacity when in frictional contact with negative triboelectric material, thereby achieving effective drug dosing release stimulated by electrostatic repulsion and satisfying the personalized needs of therapeutic effects. Additionally, the *kirigami* structures not only improves the flexible properties of the KSM-TENG patch to fit the complicated movement processes in joints but also enhances the mechanical behavior of friction. Meanwhile, the motion sensing of joints could be recorded by relative electrical output to inform patients promptly. In a treating mouse model, periodic electrostimulation accelerates drug release behaviors and prevents inflammation, which ultimately demonstrates an effective therapeutic effect for joint treatment. These features indicate that the proposed KSM-TENG patch can find applications in joint treatment and other clinical fields.

Despite these progressions, to further expand the applications of the KSM-TENG patch, much effort is still anticipated. When it comes to the combination of TENG and drug delivery systems, the clinical application in the human body should be considered. Attributed to the outstanding electrical responsiveness of TENG, cells would experience several changes in their morphology, behavior, and metabolism when exposed to electric fields. However, excessive currents and voltage pulses would generate heat when it flows through the skin, which leads to heat cell death under certain circumstances. Hence, it remains a challenge for current research to integrate the TENG with accurate temperature control systems to control drug release. Besides, another important issue is that literature demonstrated that pig skin seems to be the best model for human skin, thus most experiments were conducted on animal skins. Despite many similarities, such as epidermal thickness, membrane permeability, and lipid composition, the clinical trials in human body are still in infancy. Thus, the output performance in human trials must be evaluated to reduce in vivo immunotoxicity and biohazard. This summary will provide enlightening ideas for the present fields, it is believed that the patch is suitable for mass clinical applications.

## 4 Experiment section

### 4.1 Materials

The chimera spidroin gene consists of NT (gene bank accession number AM259067), CT (gene bank accession number JX513956), and two repeat regions (gene bank accession number AJ973155), which were synthesized by Genewiz (Suzhou, China). Silicone rubber liquids (Ecoflex) and polyurethane (PU) were obtained from Sigma–Aldrich. Lithium bromide (LiBr) and sodium carbonate (Na_2_CO_3_) were purchased from Aladdin Co., Ltd. (Shanghai, China). Ti_3_C_2_T_x_ MXene and PTFE films were purchased from 11 Technology Co. Copper foil tape was obtained from Sigma‒Aldrich Co. Rhodamine B, PTFE film, and gelatin hydrogel obtained from bovine skin were purchased from Sigma‒Aldrich Co.

### 4.2 Characterization

The optical microscope images were captured by an optical microscope (SJ-U500, SAGA). A field emission scanning electron microscope (FESEM, Zeiss Ultra Plus) was utilized to shoot the microtopography. A fluorescence microscope (DM2000, China) was employed to capture fluorescence images in this manuscript. The absorbance of the solution was measured by a spectrometer (G6860AA). The voltage values appeared in the article are instrumentally measured by an RMS multimeter (UT71D, UNI-T).

### 4.3 Fabrication of artificial spider silk solution

Using the diploid repeat sequence of chimeric spidroin MaSp1 on the cloning vector pUC57 as the template, a single point mutation was used to achieve site-directed mutation by designing appropriate primers for PCR, making the 17th and 37th positions of the diploid repeat sequence GGX of spidroin MaSp1. Position 73 X (X = glutamine Q or asparagine N) was replaced by tyrosine Y. Three mutation sites were involved in this mutation, so the required mutation sequence was obtained by using three-primer mutagenesis successively. The *Dpn*I enzyme was reacted at 37 °C for 30 min, and the obtained PCR product was digested to remove the old DNA template and methylated sequence. After screening, the mutant chimera spidroin gene was subcloned and inserted into the expression plasmid pSE380 by one-step cloning. A single colony of *E. coli* transformed with a chimeric spidroin plasmid was cultured in TB medium at 37 °C on an orbital shaker. The culture was then used to inoculate fresh TB medium, which was allowed to grow to an OD_600_ of 3–5. The culture was then induced by the addition of 1 mM IPTG and continued to grow at 30 °C for 6 h. Cells were then pelleted by centrifugation, and cell pellets were stored at −80 °C until use. The recombinant spidroin was purified by Ni-affinity chromatography followed by filtration (Millipore). Cell pellets were lysed in buffer A (6 M guanidine hydrochloride, 300 mM NaCl, 50 mM K2HPO4, pH = 8.0) for 12 h at 4 °C under constant stirring followed by centrifugation. The supernatant was loaded onto a Ni-NTA column and washed with buffer B (300 mL of NaCl, 50 mM K2HPO4, 8 M urea, pH = 8.0) with 0, 20 and 50 mM imidazole in advance. Spidroin was then eluted with buffer B containing 300 mM imidazole. All SDS‒PAGE gels were 1 mm thick and discontinuous, with a 5% stacking gel on the top and the indicated percentages of separation gels on the bottom. Samples were prepared in 4 × protein sample buffer (60 mM Tris pH 6.8, 10% glycerol, 2% SDS, 0.01% bromophenol blue, 100 μM DTT). Gels were run on Mini-PROTEAN Tetra Cells (Bio-Rad) in 1 × Tris−glycine SDS buffer (25 mM Tris base, 250 mM glycine, 0.1% w/v SDS). The protein expression level was estimated by integrating the intensity of the product band over the sum of all protein bands on the gel.

### 4.4 Fabrication of the kirigami SP-MNs patch

Laser engraving on prestretched 100% Ecoflex molds, then coating 200 μL MXene solution. After drying, the MXene solution forms to the film. Then the mold recovered to its original proportions, the SP solution was added dropwise, dried at 60 °C and formed into a needle point. The precursor solution is obtained by mixing the SP solution with 0.1 g mL^−1^ PU solution in a ratio of 7:3 by volume and is applied dropwise to the mold surface and then dried at 60 °C for 2 h to form a flexible substrate. After drying, the patch could peel off from the negative mold. Then, the kirigami structure was obtained by laser engraving at a power of 12 W.

### 4.5 Design of the kirigami KSM-TENG patch

The Kirigami SP-MNs are used as a positive friction layer, and the PTFE film is used as a negative friction layer. Both films are 1 × 3 cm^2^ in size and are pasted on top of copper foils of the same dimensions to be used as the upper and lower electrodes of the TENG.

### *4.6* In vitro *controllable drug release*

A gelatin solution (10 wt%) was placed in a petri dish and heated to solidify, and microneedles were pricked into the surface. At each time point, the patch was separated from the gelatin to stop the drug release process. Methanol: deionized water: acetic acid = 5:4:1 was used to prepare a decolorizing solution, and then the drug-diffused gelatin was immersed for 12 h. Finally, the drug diffusion was quantified by measuring the UV–Vis absorption of the decolorized solution.

### 4.7 Motion sensing experiment

The SP-MNs-TENG was fixed to different parts of the body, and then the voltage changes were measured by an RMS multimeter to record the physiological signals.

### *4.8* In vivo *therapeutic effect test*

All animal and human experiments were performed in strict accordance with the Guide for the Care and Use of Laboratory Animals and received approval from the Animal Investigation Ethics Committee of Nanjing Tech University (No. IACUC-20230301-01). Male rats (6 weeks old, weighing 300 ± 25 g) were used to model early osteoarthritis. All experimental animals were acclimatized for one week before starting the formal experiment. Different groups of rats (n = 6 per group) were injected subcutaneously with 0.1 mL complete Freund adjuvant. After injection for 24 h, the rats showed different degrees of redness and swelling and a decreased pain threshold in both paws, demonstrating that the arthritis model was successfully induced. Then, intraperitoneal injection of saline was used as a control, empty MN dressing with MTX was used as the MN group, and MN dressing with MTX was stimulated by triboelectrification as the MN-TENG group. Based on the above groups, mice were treated every 2 days for 8 days of intervention. The body weight and ankle thickness changes were observed and recorded daily. After observation, all ankle tissues were decalcified with EDTA, dehydrated in ethanol, and embedded in paraffin. Then, the sections were cut into 6 μm sections and subjected to H&E staining and Safranin O-fast green staining.

## Authors contribution

Shuhuan Li's contribution: Data curation; Formal analysis; Software; Writing - original draft and Writing - review & editing.

Suwen Cao's contribution: Conceptualization; Data curation; Formal analysis; Writing - original draft.

Huihui Lu's contribution: Conceptualization; Data curation; Formal analysis; Software; Visualization; Roles/Writing - original draft.

Bingfang He's contribution: Funding acquisition; Investigation; Resources; Software; Supervision.

Bingbing Gao's contribution: Funding acquisition; Investigation; Methodology; Project administration; Supervision; Validation.

## CRediT authorship contribution statement

**Suwen Cao:** Writing – review & editing. **Huihui Lu:** Writing – review & editing, Writing – original draft, Data curation, Conceptualization. **Bingfang He:** Supervision. **Bingbing Gao:** Supervision.

## Declaration of competing interest

The authors declare that they have no known competing financial interests or personal relationships that could have appeared to influence the work reported in this paper.

## Data Availability

Data will be made available on request.
