# Supplementary material for: Kirigami triboelectric spider fibroin microneedle patches for comprehensive joint management
Source: Mater Today Bio. 2024 Apr 4;26:101044. doi: 10.1016/j.mtbio.2024.101044 (PMC11004194; doi:10.1016/j.mtbio.2024.101044)
Supplement: Multimedia component 1 [file mmc1.docx]

Supporting Information

***Kirigami* Triboelectric Spider Fibroin Microneedle Patches for Comprehensive Joint Management**

*Shuhuan Li ^a^, Suwen Cao ^a^, Huihui Lu ^a^, Bingfang He ^a^ and Bingbing Gao ^a,*^*

^a^ College of Biotechnology and Pharmaceutical Engineering and School of Pharmaceutical Sciences, Nanjing Tech University, Nanjing 211816, China.

E-mail: [gaobb@njtech.edu.cn](mailto:gaobb@njtech.edu.cn).

Keywords: microneedles, triboelectric nanogenerator, origami, kirigami, osteoarthritis


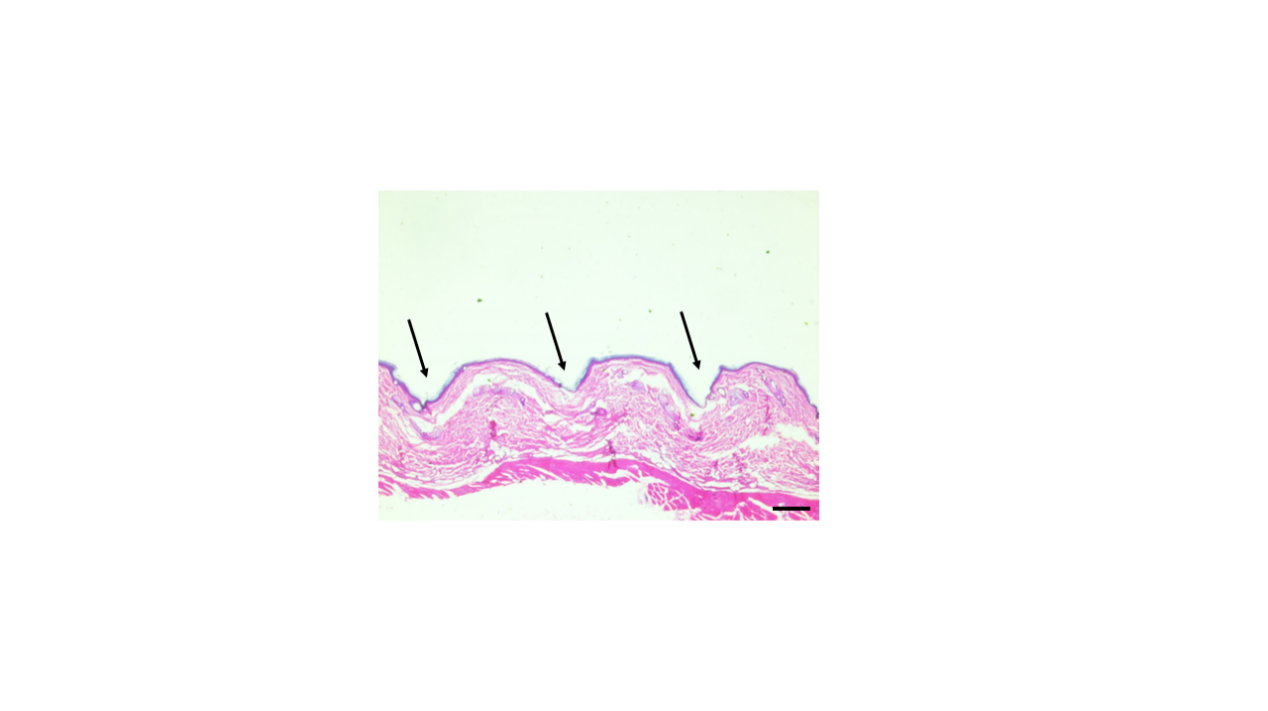


Figure S1: H&E staining of the mouse skin demonstrating the penetration of MN. Scale bar: 250 µm.

*
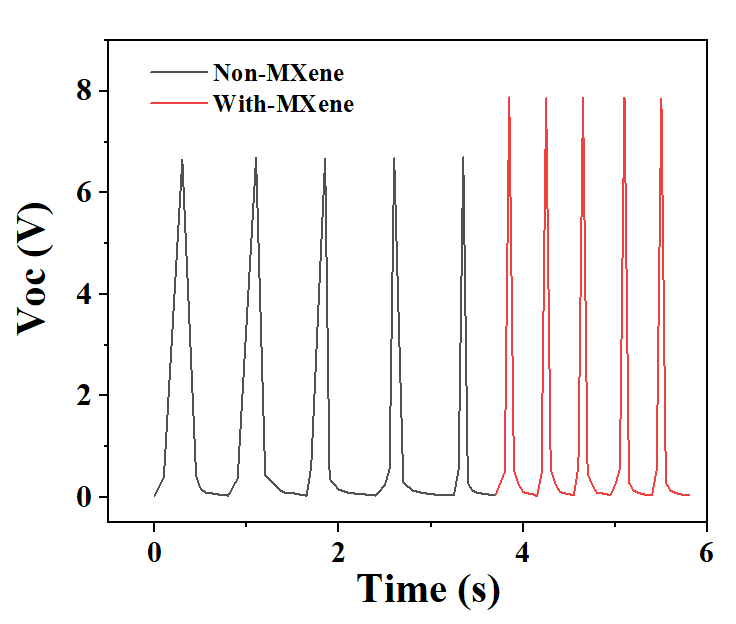
*

Figure S2: Output characteristics of generation with and without MXene solution.
